# Health-Related Quality of Life in Men with Fractures and Fear of Falling in General Population: A Cross-Sectional Study

**DOI:** 10.3390/jcm14030925

**Published:** 2025-01-30

**Authors:** Marta Zwart, Rafael Azagra-Ledesma, Miguel Ángel Díaz-Herrera, Jesus Pujol, Marc Saez, Amada Aguyé-Batista

**Affiliations:** 1 Family Medicine, Health Center Can Gibert del Pla, Institut Català de la Salut (ICS), c/San Sebastian 9, 17005 Girona, Spain; 2 Department of Medicine, Universitat de Girona (UdG), c/Emili Grahit 77, Campus Centro, 17003 Girona, Spain; 3 GROICAP, Unitat Suport a la Recerca (USR) Girona-IDIAP Jordi Gol, 17003 Girona, Spain; razagral@telefonica.net (R.A.-L.); madiaz@ambitcp.catsalut.net (M.Á.D.-H.); jpujol.lleida.ics@gencat.cat (J.P.); amyaguye@telefonica.net (A.A.-B.); 4 PRECIOSA Private Foundation for Research, Barberà del Valles, 08210 Barcelona, Spain; 5 Docencia Metropolitana Nord-Institut Català de la Salut, Universitat Autònoma de Barcelona (UAB), 08193 Barcelona, Spain; 6 Complex Wounds Unit South Metropolitan Primary Care, Institut Català de la Salut (ICS), Av/Mare de Déu de Bellvitge 3, 08907 Barcelona, Spain; 7 Department of Fundamental and Clinical Nursing, Faculty of Nursing, Universitat de Barcelona (UB), Hospitalet de Llobregat, 08907 Barcelona, Spain; 8 Hospital Universitario General de Catalunya, c/Pedro Pons 1, 08195 Barcelona, Spain; 9 Family Medicine, Health Center Balaguer, Institut Català de la Salut (ICS), c/Àngel Gimerà 22, 25600 Balaguer, Spain; 10 Department of Medicine, Universitat de Lleida (UdL), Avda/Rovira Roure 80, 25198 Lleida, Spain; 11 Research Group on Statistics, Econometrics and Health (GRECS), Universitat de Girona (UdG), c/de la Universitat de Girona 10, Campus de Montilivi, 17003 Girona, Spain; marc.saez@udg.edu; 12 CIBER of Epidemiology and Public Health (CIBERESP), 28029 Madrid, Spain; 13 Family Medicine, Health Center Granollers Vallés Oriental, Institut Català de la Salut (ICS), c/Museu 19, 08400 Granollers, Spain; 14 Departament of Medicine, Universitat Autònoma de Barcelona (UAB), Avda/Can Domènech, 08193 Bellaterra, Spain

**Keywords:** osteoporotic fracture, health-related quality of life, falls, aging

## Abstract

**Purpose:** This study aims to assess how fractures and fear of falling affect health-related quality of life (HRQoL) in men (≥50 years) across different domains. **Methods:** Design: Observational study. Setting: Primary care. Subjects: 237 men aged 50–90 years. Outcome measures: Age, frac-tures, fear of falling, EQ-5D. **Results:** A total of 122 men (51.47% of the male cohort) participated, the mean age was 69 ± 5 (≥65–74 years 26.2%, ≥75–84 years 21.3%, ≥85 years 9.8%). Poorer EQ-5D scores were observed in men ≥ 65 years with fractures in the pain domain (*p* = 0.04), while men < 65 showed better scores in mobility (*p* = 0.04), self-care (*p* = 0.04), daily activities (*p* = 0.04), and anxiety/depression (*p* = 0.01). Fear of falling significantly impacted HRQoL across all ages, with men ≥ 65 reporting worse mobility (*p* = 0.02) and higher anxiety/depression (*p* = 0.01), while men < 65 experienced less pain (*p* = 0.00). **Conclusions:** This study shows a relationship between frac-tures, fear of falling, and the perception of the various dimensions of HRQoL in older men. It highlights the need for targeted interventions and follow-up systems to monitor recovery and address fears of falling in men aged 65 and above post-fracture.

## 1. Introduction

The increase in life expectancy in developed countries has led to increased health care expenditures for age-related comorbidities. Health-related quality of life (HRQoL) based on fractures and fracture risk is one way of estimating the burden of this condition on the health care system. In Spain, annual mortality related to fractures in those over 50 is 74 per 100,000 inhabitants, with 50% due to hip fractures. Quality-Adjusted Life Years (QALYs) lost due to fragility fractures is 2.05/year per 1000 people [1,2]. The estimated costs of fragility fractures are 66% due to health status measured in QALYs, 21% due to incident fracture costs, and 11% due to long-term disability, according to data published in Europe in 2021 [2]. Less evidence is available regarding reduced quality of life in people with osteoporosis in the absence of fractures, although a relationship has been suggested both with reported osteoporosis and confirmed by bone densitometry [3] and appears to be similar to that seen in the presence of other diseases [4]. Logically, the presence of a comorbidity is a risk factor that in itself influences quality of life, as well as a fear of falling and functional frailty [5,6].

Decreased quality of life occurs with fractures in general, but especially in patients who have suffered hip and vertebral fractures [7,8,9]. In vertebral fractures, it seems that both morphometric and symptomatic vertebral fractures have an impact, especially when they occur in the lumbar spine with respect to the thoracic spine and if they are multiple vertebral fractures [5,6,10,11]. It has also been shown that the decrease occurs more significantly during the first year after a vertebral or hip fracture, and more so during the 3 months post-fracture [12]. Subsequent to fractures in these locations, during the second year after the fracture, quality of life improves, but does not return to baseline [13,14]. The impact on quality of life has been shown to be greater the more fractures the person has, and the worst estimate has been shown when the fracture is related to hospitalization, severe trauma, increased age of the patient, and lower pre-fracture quality-of-life scores [8,9,15,16].

The assessment of patients using quality-of-life questionnaires is complex because of the influence of different comorbidities on quality of life. Evidence shows that the total number of comorbidities overestimates the decline in HRQoL, and that some isolated diseases have more effect on the overall decline in quality of life than others, such as depression [10]. Another aspect that hinders their use is that general and disease-specific questionnaires offer complementary insights. Thus, for example, using the generic SF-36, reductions in certain domains of general health, vitality, physical and mental function shed little light on fracture pain, impaired body image, isolation, or subsequent mood disturbances. In contrast, using specific instruments to assess osteoporosis shows clear worsening of pain and physical function and will be more useful in individual patient assessment [17]. Finally, the costs and quality of life related to fractures show significant differences between countries and between demographic groups, including differences based on gender factors [8,17,18]. These disparities may be influenced by various factors, such as availability and accessibility of medical care, public health policies, differences in lifestyles, prevalence of osteoporosis, among others, and suggests the need for further research to better understand the disease. Historically, osteoporosis research and care has been predominantly focused on women and, in particular, in the postmenopausal context. However, osteoporosis and fragility fractures also affect men, although to a lesser extent and especially in the presence of particular characteristics, such as aging, testosterone deficiency, or the use of certain medications. The aim of this work is to evaluate the HRQoL related to fractures and fear of falling in the male population in Spain. This focus can help develop more effective and personalized strategies to address osteoporosis and fractures in men, as well as increased awareness of the importance of screening and treatment without discrimination by sex.

## 2. Materials and Methods

Design: Observational study.

Scope: Primary health care.

Main objective: To determine the loss of HRQoL in men ≥ 50 years of age with osteoporotic fracture in the Spanish population.

Participants: Men ≥ 50 years of age being treated in family medicine practices who respond to a fracture risk factor, comorbidities, and HRQoL survey during a face-to-face visit with their doctor or via telephone.

Sample selection: A random sample of 237 men was obtained from the FROCAT cohort. The detailed description of the FROCAT study has been published and includes men and women randomly selected from health centers located in the 4 provinces of Catalonia [19]. A deviation of 0.35 from the EQ-5D was used to detect a difference in variable means between fractured and non-fractured patients of 0.15 in a sample of 280 individuals (statistical significance set at 5% and 95% power). We excluded 50 (21.1%) participants who declined to participate, 19 (8%) who had died, 28 (11.8%) with a wrong telephone number, 13 (5.5%) who failed to answer 3 calls, and 5 (2.1%) who did not meet the inclusion criteria. Finally, 122 (51.5%) gave their consent to participate.

Inclusion criteria: Men ≥ 50 and ≤90 years of age who agreed to participate in the study.

Exclusion criteria: Physical or psychological difficulties that prevented participation in the study, Paget’s disease, bone cancer, terminal illnesses, or failure to respond to 3 calls made at different times.

Variables: Socio-demographic and clinical data were recorded at the time of assessment. Self-reported history of fractures was collected and confirmed through medical records. Bone Mineral Density (BMD) was assessed via dual-energy X-ray absorptiometry (DXA) at the femoral neck and lumbar spine and was expressed as T-scores. Secondary Osteoporosis was defined according to FRAX criteria [19], based on the presence of contributing conditions or treatments. Medical Conditions/Diseases were assessed using a predefined list of diagnoses, including type 2 diabetes, obesity (BMI ≥ 30 kg/m^2^), dyslipidaemia, hypertension, heart diseases, asthma, chronic obstructive pulmonary disease (COPD), stroke, chronic kidney failure, hepatopathy, knee/hip osteoarthritis, dementia, Parkinson’s disease, depression, inflammatory arthropathy, ankylosing spondylitis, and multiple sclerosis. Diagnoses were based on self-reporting or medical records. Falls were defined as any unintentional event resulting in a person coming to rest on the ground or a lower surface in the past 12 months. Use of walking aids was recorded if participants regularly used devices. Fear of falling was evaluated using a single-item question asking participants to rate their fear of falling on a scale from 1 (no fear) to 5 (always). The EQ-5D questionnaire was used to measure the health-related quality-of-life variables across five domains (mobility, self-care, activities of daily living, pain/discomfort, and anxiety/depression).

Statistical analysis: A descriptive analysis of the data was performed using percentages for categorical variables and means for normally distributed quantitative variables. For the comparison between patients with and without osteoporotic fracture we used Student’s t-test for quantitative variables and the Chi-square test for qualitative variables. Statistical significance was considered to be *p* < 0.05. Statistical analysis was performed with SPSS software, version 29.0.1.

## 3. Results

The baseline characteristics of the 122 men who participated are shown in Table 1. The mean age was 69 ± 5 years and 57.3% were 65 years of age or older, 55.7% were retired, 11.4% had a recognized disability, 25.4% were in active employment, and 7.4% were unemployed. Fifty-five (45.1%) of the men had suffered an osteoporotic fracture, 49.1% of which had occurred in the last 10 years.

**Figure 1:**
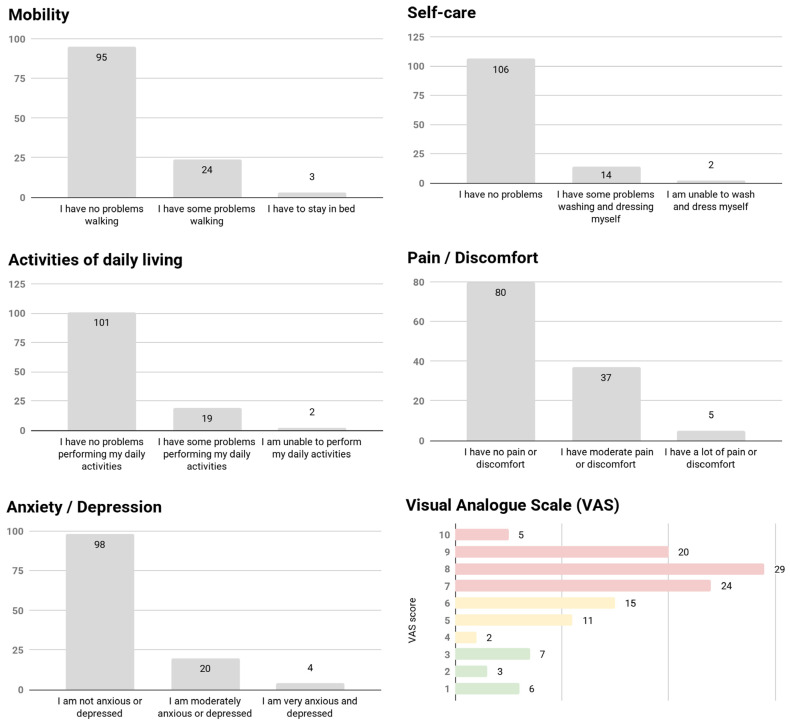
Frequencies (N) of descriptors of the EQ-5D-3L dimensions for the whole sample.

The global analysis (N = 122) of the negative results of the EQ-5D quality-of-life survey (when adding the categories “some”/“many” problems in the 5 domains) (Figure 1) showed that 27 (22.1%) described problems in the mobility domain, 16 (13.1%) in self-care, 21 (17.2%) in activities of daily living, 42 (34.4%) pain and discomfort, 24 (19.7%) anxiety and depression, and 78 (63.9%) had a Visual Analogue Scale (VAS) score ≥ 7.

Table 2 compares scores on the functional dimensions (mobility, self-care, and activities of daily living), pain and VAS, and psychological consequences (anxiety and depression) of the EQ-5D quality of life survey among men with and without a history of osteoporotic fracture. Globally, differences between fracture and no-fracture groups are less pronounced. However, when analyzed by age, men under 65 with fractures had significantly better scores compared to those ≥65 years in mobility, self-care, activities of daily living, and anxiety/depression domains and those ≥65 years with fractures had worse scores in the pain/discomfort domain. The association of worse test scores with increasing age (<65/65–74/75–84) in self-care (*p* = 0.012), activities of daily living (*p* = 0.018), pain/discomfort (*p* = 0.028), and anxiety/depression (*p* = 0.019) remained.

The stratification of the score of the different domains of the EQ-5D according to the variable fear of falling (aggregating “rarely”, “sometimes”, “often” and “always” vs. “no fear”) is shown in Table 3. Globally, significant *p*-values indicate that fear of falling strongly affects all the domains: mobility (*p* = 0.000); self-care (*p* = 0.000); activities of daily living (*p* = 0.000); pain/discomfort (*p* = 0.031); and anxiety/depression (*p* = 0.010). Subgroup analyses (<65 vs. ≥65 years) highlight a more pronounced impact on older individuals in mobility (*p* = 0.022). For anxiety/depression, both age groups (<65 and ≥65) showed significant differences between those with and without a fear of falling (*p* = 0.006 and *p* = 0.019, respectively).

## 4. Discussion

Participants in this study were randomly selected from the general population and their family doctor collected information about their medical history in person or by telephone. A relatively low percentage of the sample (2.5%) had registered bone densitometry, so we decided to make a comparison between patients with established osteoporosis, that is, with the presence of an osteoporotic fracture, and patients without a fracture.

Twenty-one percent of the men contacted declined to participate in the study. People with fractures and poorer health may have chosen not to participate, but results have also been published from studies in which patients with recent changes in health due to fragility fractures have responded more actively [9]. It is important to note that when conducting research on quality of life, various factors such as age, educational level, social class, and cultural beliefs may influence the results and may also affect the willingness to respond to this type of survey.

### 4.1. EQ-5D Questionnaire

The results of this study showed no association between a history of osteoporotic fracture when participants reported a negative EQ-5D (when adding the categories “some”/“many” problems) and compared to those with “no” problems if the results were analyzed jointly for all ages. On the other hand, when the analysis was performed in age subgroups (Table 2), those under 65 years of age with fractures had a significantly better score compared to those ≥65 years old in mobility (*p* = 0.04), self-care (*p* = 0.04), activities of daily living (*p* = 0.04), anxiety/depression (*p* = 0.01), and those ≥65 years with worse scores in pain/discomfort (*p* = 0.04). The association between worse EQ-5D scores and increasing age remained significant for self-care, activities of daily living, pain/discomfort, and anxiety/depression domains. These results suggest the importance of considering age as a relevant factor when assessing the effects of osteoporotic fractures on quality of life.

The contribution that a person’s degree of functional autonomy (mobility, self-care, and activities of daily living) makes to quality-of-life scores in older people with fractures is an aspect that merits attention in social and health policies for planning resources for health promotion in the community, giving sufficient importance to post-fracture rehabilitation, as well as to preventive measures aimed at reducing the risk of fractures in older patients. The Canadian CaMos cohort already showed a few years ago that physical function was the most impaired in fracture patients, especially after hip fractures in men over 50 years of age [20]. Also in Spain, Naves et al. published data on worse physical function in vertebral fracture and older men, coinciding with recent meta-analyses [21,22]. In the case of vertebral fractures, some studies have also found significant differences in the physical aspect in those who present vertebral deformity [11].

Mental health problems, such as depression and anxiety, can have a significant impact on the quality of life of fractured patients. One study focused on recent hip fractures found that lower scores on geriatric depression scales were associated with poorer quality of life, while patients taking antidepressants reported better quality of life [7]. These findings highlight the importance of evaluating not only the fractures themselves, but also other factors such as cognitive status, depression, and treatment of mental health issues in patients with hip fractures.

Fractures can cause chronic pain over time, as in vertebral crushing or poorly consolidated hip or wrist fractures. Previous European studies have highlighted that pain is the most affected quality-of-life dimension in men with fractures, especially in older individuals and those with at least two fractures [15]. In vertebral fractures, acute back pain has the greatest impact on quality of life in men, followed by self-care (17%), mobility (16%), and daily activities (10%) [23,24]. This means that it is necessary not only to treat the fracture itself, but also to provide comprehensive pain management, including physiotherapy, pharmacological treatment, psychological support, and other supportive measures.

Complementing this information, results have been published showing that there are fewer differences in EQ-5D scores between older patients with osteoporosis and/or fractures and the general population compared to younger individuals [25]. This decrease in differences could be attributed to several factors, including quality-of-life expectations among different age groups. Younger individuals, generally, may have higher quality-of-life expectations compared to older individuals, and may have experienced a life with greater physical functionality, mobility, and less experience with chronic diseases or disabilities, which could lead them to perceive more significantly the impact of osteoporosis or fractures on their quality of life. On the other hand, older individuals may have experienced a progressive decline in health and functionality as they age, which could lead them to have quality-of-life expectations that are more in line with their current condition. This does not mean that fractures or osteoporosis do not have an impact on their quality of life, but that it may be perceived as part of their natural aging process or previous experiences with health. These findings underscore the importance of considering individual expectations and perceptions when assessing quality of life due to their relationship to osteoporosis and osteoporotic fractures.

### 4.2. Fear of Falling

Several studies have shown that fear of falling significantly impacts the quality of life in older adults, often reducing physical activity and initiating a cycle of functional decline [4]. People affected by fractures fall into a cascade of sedentary lifestyles and sarcopenia derived from reduced mobility, which leads them to walk with a certain instability, increasing the risk of falling and causing loss of autonomy due to the fear of falling and fractures [26]; in short, a dependence on their caregivers or the health care system. When assessing the fear of falling in this study population, we observed that it scored significantly worse on the EQ-5D questionnaire in all participants who reported fear of falling (Table 3). In the age-stratified analysis, it was observed that those participants ≥ 65 years old who had a fear of falling showed worse scores in mobility (*p* = 0.022) and anxiety/depression (*p* = 0.019). Conversely, the participants < 65 years old who had no fear of falling showed a better score in pain/discomfort (*p* = 0.003) and anxiety/depression (*p* = 0.006). These results are in line with negative results observed in other European studies between fear of falling and alterations in the physical and mental domains of the HRQoL questionnaires, especially in older people [27].

The analysis of the fear of falling in consultations is often underestimated and the results presented are a strength of this work because they help to understand how this fear affects the male population, providing a valuable and necessary perspective to implement personalized strategies to help mitigate this fear [28]. The findings related to falls from a gender perspective become more relevant in light of recent meta-analyses that highlight the differential effect between previous fall and fracture risk, with predictive values being higher in men than in women [29].

The most important limitation of this work was the small number of subjects who agreed to participate in the study. This implied low statistical power, i.e., a reduced probability of rejecting the null hypothesis when it was false, which would have led to finding very few or even no contrasts with a low *p*-value. However, we found quite a few contrasts with a *p*-value of less than 0.05. This suggests that, even with a small sample size, there were many differences that could not be attributed to chance.

Regarding the study population and future research, it would be important to collect more detailed information on bone mineral density in the general population for a more accurate assessment of quality of life in patients with osteoporosis at earlier stages of the disease, when they have not yet experienced fractures. It is also important to keep in mind that the perception of quality of life may vary over time and be influenced by different circumstances, medical treatments, lifestyle changes, or health conditions. Rohde et al. observed worse results in quality-of-life assessment at the 2-year follow-up compared to the 1-year follow-up in the same cohort of patients with osteoporotic fractures [9]. In the first year after a fracture, individuals may be experiencing an initial recovery phase in which they see improvements in their mobility, autonomy, and general well-being. Yet, as time goes on, complications such as chronic pain or persistent limitations may develop, which affect their long-term quality of life. Further work focusing on quality-of-life assessment over a prolonged period would allow for detailed analysis of HRQoL at different stages of recovery and adaptation to fracture, and by using morbidity clusters to identify trends in health status in order to gain a more complete picture of the patient’s situation.

## 5. Conclusions

In summary, the findings of this study suggest that there is a relationship between the fear of falling and fracture history and the perception of health-related quality of life in men, especially at older ages. Addressing the fear of falling and taking measures to prevent falls may be crucial not only to reduce the risk of fractures, but also to improve the perception of health and quality of life in these vulnerable populations, regardless of whether or not they have diseases, by seeking a more positive state of wellbeing. Also, the rehabilitation programs tailored to regain mobility and reduce pain after fractures can make a significant difference in their recovery trajectory

## Figures and Tables

**Table 1:** Baseline characteristics.

	Men N = 122
Age (years) (mean, SD)	69 ± 5
Age < 65 (N, %)	52 (42.6%)
Age ≥ 65–74 (N, %)	32 (26.2%)
Age ≥ 75–84 (N, %)	26 (21.3%)
Age ≥ 85 (N, %)	12 (9.8%)
Retired (N, %)	68 (55.7%)
Disability (N, %)	14 (11.4%)
Active employment (N, %)	31 (25.4%)
Unemployed (N, %)	9 (7.4%)
Personal history fracture (N, %)	55 (45.1%)
Comorbidity ≥ 3 (N, %)	31 (25.4%)
Falls in previous year (N, %)	29 (23.7%)
≥2 Falls in previous year (N, %)	8 (6.5%)
Use of walking aids (N, %)	9 (7.3%)
Bone densitometry tested, yes (N, %)	3 (2.5%)

SD: standard deviation; N: cases.

**Table 2:** Comparison of EQ-5D questionnaire results between groups with and without fractures, for all ages and by age subgroups: negative results versus no reported change (unaltered’) in quality-of-life scores.

		Age (Year)	History of Fracture and/or Fracture in the Last 10 Years		*p* <65 vs. ≥65
			Yes, N (%)	No, N (%)	
Mobility	No change		42 (76.4%)	52 (77.6%)	0.870
	Change		13 (23.6%)	15 (22.4%)	
	No change	<65	28 (66.7%)	44 (84.6%)	0.041
		≥65	14 (33.3%)	8 (15.4%)	
	Change	<65	5 (38.5%)	7 (46.7%)	0.662
		≥65	8 (61.5%)	8 (53.3%)	
Self-care	No change		49 (89.1%)	57 (85.1%)	0.513
	Change		6 (10.9%)	10 (14.9%)	
	No change	<65	32 (65.3%)	47 (82.5%)	0.043
		≥65	17 (34.7%)	10 (17.5%)	
	Change	<65	1 (16.7%)	4 (40.0%)	0.330
		≥65	5 (83.3%)	6 (60.0%)	
Activities of daily living	No change		47 (85.5%)	54 (80.6%)	0.479
	Change		8 (14.5%)	13 (19.4%)	
	No change	<65	31 (66.0%)	45 (83.3%)	0.044
		≥65	16 (34.0%)	9 (16.7%)	
	Change	<65	2 (25.0%)	6 (46.2%)	0.332
		≥65	6 (75.0%)	7 (53.8%)	
Pain/discomfort	No change		37 (67.3%)	43 (64.2%)	0.720
	Change		18 (32.7%)	24 (35.8%)	
	No change	<65	25 (67.6%)	33 (76.7%)	0.359
		≥65	12 (32.4%)	10 (23.3%)	
	Change	<65	8 (44.4%)	18 (75.0%)	0.044
		≥65	10 (55.6%)	6 (25.0%)	
Anxiety/ depression	No change		44 (80.0%)	54 (80.6%)	0.934
	Change		11 (20.0%)	13 (19.4%)	
	No change	<65	25 (56.8%)	43 (79.6%)	0.015
		≥65	19 (43.2%)	11 (20.4%)	
	Change	<65	8 (72.7%)	8 (61.5%)	0.562
		≥65	3 (27.3%)	5 (38.5%)	

N: cases.

**Table 3:** Comparison of EQ-5D questionnaire results between groups with and without a fear of falling, for all ages and by age subgroups: negative results versus no reported change (unaltered’) in quality-of-life scores.

		Age (Years)	Fear of Falling			
			Yes, N (%)	No, N (%)	*p*	*p*
Mobility	No change		13 (10.7%)	81 (66.4%)	0.000	
	Change		14 (11.5%)	14 (11.5%)		
	No change	<65	8 (61.5%)	64 (79%)		0.167
		≥65	5 (38.5%)	17 (21%)		
	Change	<65	3 (21.4%)	9 (64.3%)		0.022
		≥65	11 (78.6%)	5 (35.7%)		
Self-care	No change		16 (13.1%)	90 (73.8%)	0.000	
	Change		11 (9.0%)	5 (4.1%)		
	No change	<65	9 (56.3%)	70 (77.8%)		0.069
		≥65	7 (43.8%)	20 (22.2%)		
	Change	<65	2 (18.2%)	3 (60%)		0.094
		≥65	9 (81.8%)	2 (40%)		
Activities of daily living	No change		14 (11.5%)	87 (71.3%)	0.000	
	Change		13 (10.7%)	8 (6.6%)		
	No change	<65	8 (57.1%)	68 (78.2%)		0.091
		≥65	6 (42.9%)	19 (21.8%)		
	Change	<65	3 (23.1%)	5 (65.5%)		0.071
		≥65	10 (76.9%)	3 (37.5%)		
Pain/discomfort	No change		13 (10.7%)	67 (54.9%)	0.031	
	Change		14 (11.5%)	28 (23%)		
	No change	<65	5 (38.5%)	53 (79.1%)		0.003
		≥65	8 (61.5%)	14 (20.9%)		
	Change	<65	6 (42.9%)	20 (71.4%)		0.072
		≥65	8 (57.1%)	8 (28.6%)		
Anxiety/depression	No change		17 (13.9%)	81 (66.4%)	0.010	
	Change		10 (8.2%)	14 (11.5%)		
	No change	<65	7 (41.2%)	61 (75.3%)		0.006
		≥65	10 (58.8%)	20 (24.7%)		
	Change	<65	4 (40.0%)	12 (85.7%)		0.019
		≥65	6 (60.0%)	2 (14.3%)		

N: cases.

## Data Availability

The datasets used and/or analyzed during the current study are available from the corresponding author on reasonable request.

## Abbreviations

EQ-5D	European Quality of Life Five-Dimensional descriptive system questionnaire
HRQoL	Health-related Quality of Life
QALY	Quality-Adjusted Life Years
VAS	Visual Analogue Scale
